# Primary aldosteronism in patients with acute stroke: prevalence and diagnosis during initial hospitalization

**DOI:** 10.1186/s12883-016-0701-5

**Published:** 2016-09-17

**Authors:** Yosuke Miyaji, Yuichi Kawabata, Hideto Joki, Shunsuke Seki, Kentaro Mori, Tomoya Kamide, Akira Tamase, Hiroshi Shima, Motohiro Nomura, Yoshihisa Kitamura, Hirotatsu Nakaguchi, Taichi Minami, Tetsuji Tsunoda, Mayuko Sasaki, Masayo Yamada, Fumiaki Tanaka

**Affiliations:** 1 Department of Neurology and Stroke Medicine, Yokohama Sakae Kyosai Hospital, Yokohama, Japan; 2 Department of Neurosurgery and Stroke Medicine, Yokohama Sakae Kyosai Hospital, Yokohama, Japan; 3 Department of Metabolism and Endocrinology, Yokohama Sakae Kyosai Hospital, Yokohama, Japan; 4 Department of Neurology and Stroke Medicine, Yokohama City University Graduate School of Medicine, 3-9 Fukuura, Kanazawa-ku, Yokohama, 236-0004 Japan

**Keywords:** Stroke, Primary aldosteronism, Secondary hypertension, Risk factor, Intracranial hemorrhage, Ischemic stroke

## Abstract

**Background:**

Hypertension is the prime risk factor for stroke, and primary aldosteronism (PA) is the most common cause of secondary hypertension. The prevalence of PA in stroke patients has never been reported. The aim of this study was to elucidate the prevalence of PA.

**Methods:**

A total of 427 consecutive patients with acute stroke were prospectively enrolled for this study. The screening tests were performed at the initial visit and a week after admission by measuring plasma aldosterone concentration and plasma renin activity. The rapid adrenocorticotropic hormone (ACTH) test was performed as the confirmatory test when both screening tests were positive. The primary endpoint was a final diagnosis of PA.

**Results:**

The sensitivity of the dual screening system for the diagnosis of PA was 88.2 %, and PA was finally diagnosed in 4.0 % of acute stroke patients and in 4.9 % of stroke patients with a history of hypertension. Patients with PA were less likely to be male and have diabetes, and they had higher blood pressure at the initial visit, lower potassium concentration, and more intracerebral hemorrhage. The rapid ACTH test was performed safely even in acute stroke patients.

**Conclusions:**

The prevalence of PA is not low among acute stroke patients. Efficient screening of PA should be performed particularly for patients with risk factors.

**Trial registration:**

UMIN-CTR; UMIN000011021. Trial registration date: June 23, 2013 (retrospectively registered).

## Background

Hypertension is the prime risk factor for stroke [1], and treatment of hypertension is highly effective in the prevention of stroke [2, 3]. Secondary hypertension is identified in a relatively small proportion of adult patients with hypertension. However, particular attention should be paid to this condition, because the hypertension can be cured by appropriate specific treatment. Primary aldosteronism (PA) is caused by the autonomous secretion of aldosterone from adrenocortical lesions and is associated with hypertension and hypokalemia. PA has attracted much attention in recent years because PA is more frequent than previously recognized, occurring in 3–10 % of hypertensive patients [4–6]. PA patients have a higher rate of vascular complications [7, 8] and a poorer long-term prognosis than patients with essential hypertension [9]. Interestingly, the occurrence of cerebrovascular comorbidities in patients with PA is reported to be independent of hypertension and hypokalemia [8, 10]. Therefore, aside from vascular injury due to hypertension, the direct action of aldosterone on the mineralocorticoid receptor may cause increased oxidative stress and collagen remodeling, which results in endothelial dysfunction and fibrosis in the blood vessels [11].

Most PA patients are successfully treated by unilateral adrenalectomy or with mineralocorticoid receptor antagonists [12, 13], and the blood pressure becomes normalized [14, 15]. Furthermore, some reports have demonstrated improvement of left ventricular hypertrophy, renal function, and the cardiovascular prognosis with PA treatment [16–18].

Although early diagnosis and treatment of PA are important for these reasons, stroke patients are not included among the subjects for aggressive PA screening in the guidelines [12, 13, 19], and there have been no previous reports about the screening of PA in patients with acute stroke. The aim of this prospective study was to elucidate the prevalence and risk factors of PA in patients with acute stroke.

## Methods

### Study population

Consecutive patients with acute stroke including transient ischemic attack (TIA) who were admitted to Yokohama Sakae Kyosai Hospital between April 2013 and March 2014 were prospectively enrolled for this study. The definition for “acute stroke inpatient” in this study was “emergency admission” and “the requirement of acute management of stroke”. The time from the onset of stroke to admission was 0.6 ± 1.1 days (mean ± SD; range, 0–6; median, 0). In addition to clinical evaluation, including a history of vascular risk factors, all patients underwent brain computed tomography and/or magnetic resonance imaging, chest X-ray, electrocardiography, and standard blood tests. The vascular risk factors examined were hypertension, diabetes mellitus, dyslipidemia, history of smoking, and habitual drinking. The Trial of Org 10172 in Acute Stroke Treatment criteria were used for the classification of ischemic stroke [20]. TIA was defined as a transient episode of neurological dysfunction without evidence of acute cerebral infarction on neuroimaging [21].

The study was done in accordance with the principles of the Helsinki declaration and was approved by The Ethics Committee of Yokohama Sakae Kyosai Hospital (approval number: 2013041601). Written informed consent was obtained from all patients in compliance with the committee’s requirements. This study was registered with the University Hospital Medical Information Network Clinical Trials Registry (UMIN-CTR, identifier: UMIN000011021).

### Screening test

For the screening of PA, plasma aldosterone concentration (PAC) and plasma renin activity (PRA) were measured simultaneously, and the PAC to PRA ratio (ARR) was calculated, as previously described [22]. The criteria for a positive screening test for PA were ARR ≥200 and PAC ≥12 ng/dL [13]. The screening tests were performed twice at the initial visit and about a week after the admission, and the confirmatory test was performed when both screening tests were positive. Although patients who were positive only on a single screening test were in principle excluded from the confirmatory test, exceptions were made for some patients because of the potential effect of prehospital use or new administration of antihypertensive drugs.

### Confirmatory test

The rapid adrenocorticotropic hormone (ACTH) test was performed as the confirmatory test, and patients with a positive result were given the definitive diagnosis of PA. The mechanism of the rapid ACTH test is confirmation of the hyperactivation of aldosterone by ACTH stimulation in cases with aldosterone hypersecretion [23]. The test was performed as follows. The patient was asked to lie on a bed in the supine position for 30 min in the morning fasting state, and blood samples were collected 30 and 60 min after the intravenous injection of 0.25 mg ACTH. If the ratio of the maximal PAC to the simultaneously-measured cortisol was ≥8.5, PA was diagnosed [24]. The primary endpoint was a final diagnosis of PA.

### Statistical analysis

Data were analyzed with the use of Excel (version 2010; Microsoft Corp, Redmond, WA) and Dr. SPSS II (SPSS Inc, Chicago, IL). Demographic characteristics were compared using Mann-Whitney’s U-test for continuous variables, and the Chi-square test or Fisher’s exact test for categorical variables. The differences were considered significant with a *P* value <0.05.

## Results

### Patients’ baseline characteristics

**Fig. 1:**
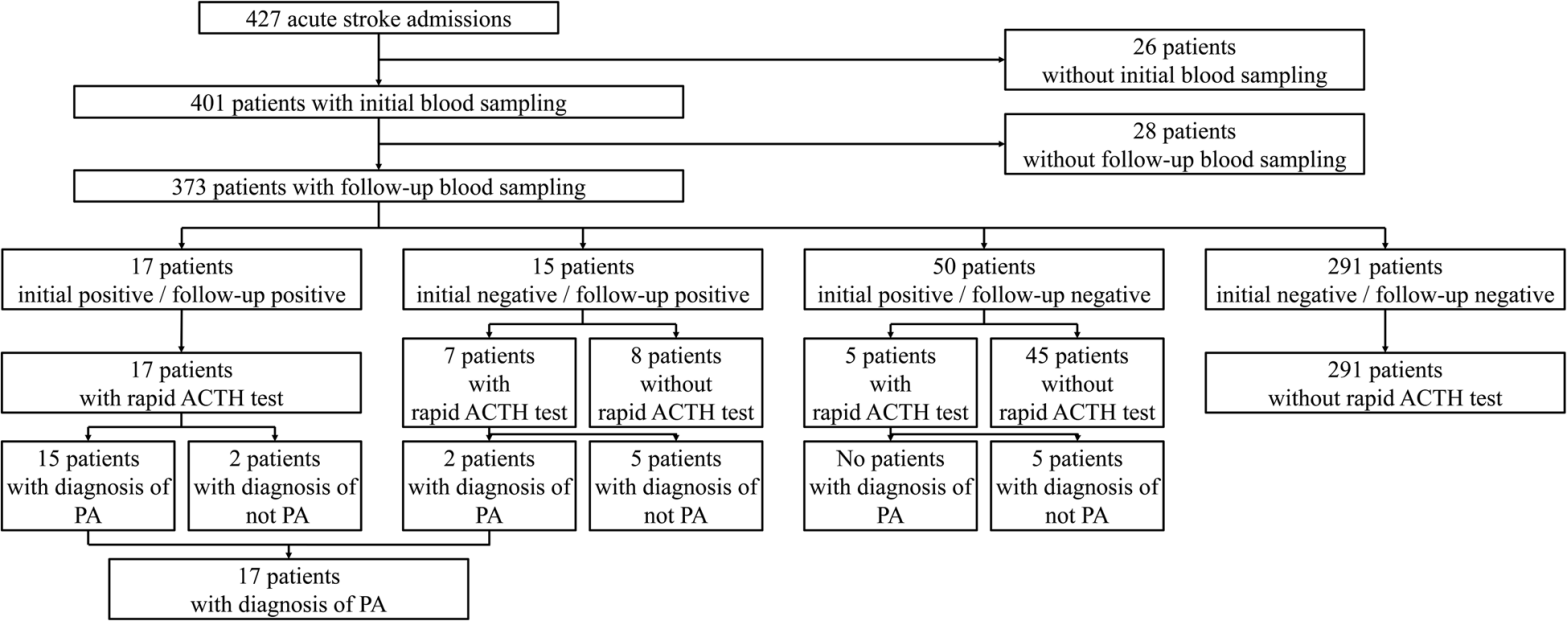
Flow chart of the study population

A total of 427 patients with acute stroke were enrolled during the study period. No patients were diagnosed with PA before admission. The demographic characteristics of the patients are shown in Table 1. The mean age was 74.3 ± 11.9 years, and 56.7 % of the patients were male. Hypertension was recognized in 67.4 % of the patients, and 55.3 % were treated with antihypertensive drugs. The mean blood pressure at the initial visit was 162.8 ± 31.6/89.7 ± 20.2 mmHg. The subtypes of stroke were: ischemic stroke (60.0 %), intracerebral hemorrhage (24.8 %), subarachnoid hemorrhage (8.9 %), and TIA (6.3 %). The study flow chart is shown in Fig. 1.

**Table 1:** Patients’ demographic characteristics

	All patients	Patients		*P* value
		with PA	without PA	
	(*n* = 427)	(*n* = 17)	(*n* = 356)	
Age, y	74.3 ± 11.9	70.6 ± 10.3	74.6 ± 11.8	0.095^a^
Male	242 (56.7 %)	5 (29.4 %)	212 (59.6 %)^d^	0.014^b^
Hypertension	288 (67.4 %)	14 (82.4 %)	245 (68.8 %)	0.237^b^
Diabetes	75 (17.6 %)	0 (0 %)	70 (19.6 %)^d^	0.027^c^
Dyslipidemia	120 (28.1 %)	3 (17.6 %)	104 (29.2 %)	0.230^c^
History of smoking	122 (28.6 %)	2 (11.8 %)	108 (30.3 %)	0.101^b^
Habitual drinking	140 (32.8 %)	4 (23.5 %)	125 (35.1 %)	0.327^b^
Initial systolic blood pressure, mmHg	162.8 ± 31.6	179.9 ± 26.1^d^	162.0 ± 31.6	0.012^a^
Initial diastolic blood pressure, mmHg	89.7 ± 20.2	101.8 ± 15.7^d^	89.2 ± 20.3	0.003^a^
Potassium, mmol/L	4.1 ± 1.1	3.7 ± 0.4	4.1 ± 0.5^d^	0.001^a^
Stroke subtypes				
Ischemic stroke	256 (60.0 %)	7 (41.2 %)	225 (63.2 %)	0.067^b^
Large artery atherosclerosis	69 (16.2 %)	1 (5.9 %)	62 (17.4 %)	0.185^c^
Cardioembolism	93 (21.8 %)	3 (17.6 %)	81 (22.8 %)	0.443^c^
Small vessel occlusion	38 (8.9 %)	0 (0 %)	34 (9.6 %)	0.190^c^
Undetermined pathogenesis	56 (13.1 %)	3 (17.6 %)	48 (13.5 %)	0.418^c^
Intracerebral hemorrhage	106 (24.8 %)	8 (47.1 %)^d^	86 (24.2 %)	0.038^c^
Subarachnoid hemorrhage	38 (8.9 %)	0 (0 %)	24 (6.7 %)	0.315^c^
Transient ischemic attack	27 (6.3 %)	2 (11.8 %)	21 (5.9 %)	0.282^c^
Antihypertensive medication	236 (55.3 %)	9 (52.9 %)	203 (57.0 %)	0.740^b^
Calcium antagonist	166 (38.9 %)	9 (52.9 %)	134 (37.6 %)	0.233^b^
ARB / ACEI	123 (28.8 %)	2 (11.8 %)	87 (24.4 %)	0.101^b^
Direct renin inhibitor	1 (0.2 %)	0 (0 %)	1 (0.3 %)	0.954^c^
Diuretic	63 (14.8 %)	1 (5.9 %)	45 (12.6 %)	0.227^c^
Beta blocker	44 (10.3 %)	0 (0 %)	24 (6.7 %)	0.132^c^
Others	8 (1.9 %)	0 (0 %)	7 (2.0 %)	0.754^c^

Values in the table are means ± SD or Nos (%)

*Abbreviations*: *ARB* angiotensin receptor blocker; *ACEI* angiotensin-converting enzyme inhibitor

*P* value calculated by ^a^Mann-Whitney’s U-test, ^b^Chi-square test, or ^c^Fisher’s exact test.  ^d^indicates significantly higher or more frequent

### Screening test

The initial screening test was performed in 401 patients; it was not completed in 26 patients due to failure to sample PAC and PRA (Fig. 1). The follow-up screening test was not performed in 28 more patients because they were discharged or transferred from our hospital or died before the test. The follow-up screening test was performed in a total of 373 patients, 6.8 ± 3.5 days after the hospitalization on average. The initial and follow-up screening tests were positive in 67 patients (18.0 %) and 32 patients (8.6 %), respectively. All of the 17 patients with positive results on both screening tests underwent the confirmatory test. There were 15 patients with a negative initial test and a positive follow-up test, and 7 of them underwent the confirmatory test because they might have been false-negative on initial screening due to the prehospital use of antihypertensives. There were 50 patients with a positive initial test and a negative follow-up test, and 5 of them underwent the confirmatory test because they might have been false-negative on the follow-up screening due to the start of antihypertensives after admission. A total of 291 patients (78.0 %) showed negative results on both tests.

### Confirmatory test

A total of 29 patients underwent the confirmatory test. Fifteen of the 17 patients (88.2 %) with positive results on both screening tests showed a positive result on the confirmatory test. Two of 7 patients (28.6 %) with a negative initial test and a positive follow-up test and none of the five (0.0 %) patients with a positive initial test and a negative follow-up test showed positive results on the confirmatory test. No adverse events were observed in relation to the confirmatory test using intravenous injection of ACTH.

### Prevalence of primary aldosteronism in patients with acute stroke

Seventeen patients (14 with a history of hypertension) were finally diagnosed with PA, and they constituted 4.0 % of the total participants and 4.9 % of the patients with a history of hypertension. Among the 373 patients who underwent both screening tests, the prevalence of PA was 4.6 % of the total and 5.5 % of the hypertensive patients.

### Characteristics of stroke patients with primary aldosteronism

The demographic characteristics of the patients with and without PA are shown in Table 1. The patients with PA included fewer males (*P* = 0.014) and fewer with diabetes (*P* = 0.027), and they had higher blood pressure at the initial visit (systolic *P* = 0.012, diastolic *P* = 0.003), lower potassium concentration (*P* = 0.001), and more intracerebral hemorrhage (*P* = 0.038). Although we found no significant differences in ischemic stroke subtypes between patients with and without PA, all cardioembolic strokes were based on non-valvular atrial fibrillation and the culprit artery for atherothrombotic stroke was the internal carotid artery in PA patients. The topographies of hemorrhages in patients with PA were thalamus for 5 patients, and putamen, pons, and cerebellum for each one of the remaining 3 patients. The mechanism of hemorrhagic stroke in all PA patients was hypertensive intracerebral hemorrhage. Only 5 patients (29.4 %) had potassium concentrations below the lower limit of normal (3.5 mmol/L).

## Discussion

In the present study, the prevalence rate of PA in patients with acute stroke was 4.0 % of all patients and 4.9 % of patients with hypertension, which is comparable to that in previous reports of 3–10 % in the general hypertensive population [4–6]. This study clearly demonstrated for the first time that the rate of PA among stroke patients is considerable, which has not been described previously. However, this prevalence rate is likely to be an underestimate, because confirmatory tests necessary for the definite diagnosis of PA were not performed in all 82 patients who were positive on at least one screening test. Therefore, the real prevalence rate of PA in stroke patients might be higher than that reported in this study.

The ARR of the screening tests may be easily affected by various factors, especially in stroke patients. A false-positive ARR elevation at the initial blood sampling might be caused by the elevation of PRA and PAC due to dehydration and by the decrease of PRA due to high blood pressure in the acute stroke phase. Therefore, confirmatory tests were not done when initial positive and follow-up negative results were obtained on the screening tests. However, the confirmatory test was performed in 5 patients (Fig. 1) who were administered antihypertensive agents after the stroke because such treatment may decrease ARR and lead to a false-negative result on the follow-up test. However, none of these patients (0.0 %) were finally diagnosed with PA.

As for the interpretation of initial negative and follow-up positive results on the screening tests, a false-negative result might have been due to prehospital administration of antihypertensive agents, and a false-positive ARR elevation on the follow-up screening test might have been caused by the effect of stopping or switching antihypertensive drugs after the stroke. Therefore, 7 patients who fit the above situation were selected, and the confirmatory test was performed (Fig. 1); of these, 2 patients (28.6 %) were diagnosed with PA.

Of the 17 patients with positive results on the initial and follow-up screening tests, 15 (88.2 %) were diagnosed with PA (Fig. 1), suggesting that dual-positive results on two screening tests are highly reliable as compared to a single-positive result (0.0 % and 28.6 % on initial and follow-up tests, respectively), which is consistent with the recommendation of the dual screening system in the guidelines [13, 19]. The initial screening test was less reliable than the follow-up test, thus the first test might be better to be performed later than at the first visit. However, we have no data to verify this speculation. Because the confirmatory test for the definitive diagnosis of PA needs the test drug (ACTH) and more sets of laboratory tests, we believe the dual screening system may be easy and cost-effective to perform in acute phase of stroke.

Screening tests for PA are recommended as much as possible for all patients with hypertension [13]. Because patients presenting with PA are reported to have a higher frequency of cerebrovascular events than patients with essential hypertension [8], it is important to not overlook treatable PA for the prevention of recurrence in stroke patients. However, several limitations should also be taken into account when screening for PA in stroke patients. First, the strict screening protocol, such as cessation of antihypertensive drugs [13], cannot be applied to stroke patients because treatment for stroke in the acute phase has a higher priority than the diagnosis of PA. Second, the older age and the low level of activities of daily living in stroke patients generally make it difficult to perform adrenalectomy even if the diagnosis of PA were made. In fact, the 17 patients diagnosed with PA in the present study neither underwent invasive adrenal venous sampling for the subtype and localization diagnosis nor adrenalectomy due to the reasons described above or the patients’ refusal of further examination. Third, the cost-effectiveness of the screening for PA in all stroke patients should also be considered.

Considering these special circumstances in stroke patients, intensive screening would be realistic in younger patients at high risk for PA. The results of the present study demonstrated that female sex, absence of diabetes, high blood pressure at the initial visit (including poorly-controlled blood pressure), lower potassium level, and intracerebral hemorrhage were the risk factors for PA (Table 1). Although small vessel occlusion is most related to hypertension among ischemic strokes [25], we found no PA patients with this subtype. This result either may be incidental, or may indicate that the effect of PA on the occurrence of ischemic stroke largely depends on the direct action of aldosterone on the mineralocorticoid receptor.

We emphasize that it is important to recognize the high prevalence of PA in stroke patients and that intensive and efficient screening should be performed in patients with several of the risk factors for PA identified in this study.

## Conclusions

In acute stroke, PA was diagnosed in 4.0 % of total patients and in 4.9 % of patients with a history of hypertension. In screening for PA, a dual screening system including initial and follow-up tests seemed reliable compared to a single screening system, because 88.2 % of patients positive on both tests were confirmed to have PA. The rapid ACTH test for the confirmation of PA was performed safely. It is important to pay more attention to the possibility of the existence of PA in acute stroke patients, and efficient screening of PA should be performed particularly for young patients, considering the risk factors clarified in this study.

## Abbreviations

ACTH: Adrenocorticotropic hormone
ARR: Plasma aldosterone concentration to plasma renin activity ratio
PA: Primary aldosteronism
PAC: Plasma aldosterone concentration
PRA: Plasma renin activity
TIA: Transient ischemic attack
